# A phase III randomized-controlled, single-blind trial to improve quality of life with stereotactic body radiotherapy for patients with painful bone metastases (ROBOMET)

**DOI:** 10.1186/s12885-019-6097-z

**Published:** 2019-09-04

**Authors:** Carole Mercier, Piet Dirix, Piet Ost, Charlotte Billiet, Ines Joye, Peter Vermeulen, Yolande Lievens, Dirk Verellen

**Affiliations:** 1 Department of Radiation Oncology, Iridium Kankernetwerk, Oosterveldlaan 22, Wilrijk, B-2610 Antwerp, Belgium; 2 Molecular Imaging, Pathology, Radiotherapy & Oncology (MIPRO), University of Antwerp, Edegem, Antwerp, Belgium; 3 Department of Radiotherapy, Ghent University Hospital, Ghent, Belgium; 4 Translational Cancer Research Unit, Oncologisch Centrum GZA, Wilrijk, Antwerp, Belgium; 5 Vrije Universiteit Brussel (VUB), Brussels, B-1090 Belgium

**Keywords:** Stereotactic body radiotherapy, Bone metastases, Spinal metastases, Pain

## Abstract

**Background:**

Bone metastases represent an important source of morbidity in cancer patients, mostly due to severe pain. Radiotherapy is an established symptomatic treatment for painful bone metastases, however, when conventional techniques are used, the effectiveness is moderate. Stereotactic body radiotherapy (SBRT), delivering very high doses in a limited number of fractions in a highly conformal manner, could potentially be more effective and less toxic.

**Methods:**

This is a phase III, randomized-controlled, single-blind, multicenter study evaluating the response rate of antalgic radiotherapy for painful bone metastases and the acute toxicity associated with this treatment. A total of 126 patients will be randomly assigned to receive either the standard schedule of a single fraction of 8.0 Gy delivered through three-dimensional conformal radiotherapy or a single fraction of 20.0 Gy delivered through SBRT. Primary endpoint is pain response at the treated site at 1 month after radiotherapy. Secondary endpoints are pain flare at 24–48-72 h after radiotherapy, duration of pain response, re-irradiation need, acute toxicity, late toxicity, quality of life and subsequent serious skeletal events. In a supplementary analysis, patient-compliance for a paper-and-pencil questionnaire will be compared with an electronic mode.

**Discussion:**

If a dose-escalated approach within the context of single fraction stereotactic body radiotherapy could improve the pain response to radiotherapy and minimize acute toxicity, this would have an immediate impact on the quality of life for a large number of patients with advanced cancer. Potential disadvantages of this technique include increased pain flare or a higher incidence of radiation-induced fractures.

**Trial registration:**

The Ethics committee of the GZA Hospitals (B099201732915) approved this study on September 4th 2018. Trial registered on Clinicaltrials.gov (NCT03831243) on February 5th 2019.

## Background

Regrettably, a large proportion of cancer patients will ultimately develop systemic disease. Bone metastases are a common manifestation of distant relapse from many types of solid tumors, especially those arising in the lung, breast and prostate. They represent an important source of morbidity in these patients, mostly due to severe pain. Furthermore, they can cause hypercalcemia, pathologic fractures and spinal cord compression, all of which can significantly compromise quality of life. The goals of palliative radiotherapy of bone metastases are pain relief, preservation of function, and maintenance of skeletal integrity. Radiotherapy is an established symptomatic treatment for painful bone metastases. A common and convenient schedule uses a single dose of 8 Gy [1]. Several other fractionation schedules, using moderate dose escalation (5 × 4.0 Gy or 10-13 × 3.0 Gy), have been investigated. However, so far, none has demonstrated superiority to a single 8 Gy fraction [2]. A large meta-analysis by Rich et al. showed overall response rates of 61% versus 62% for single fraction and multiple fraction regimens [3]. Complete responses were seen in 23% versus 24% of patients. A drawback of single 8 Gy fraction treatment is a consistently higher retreatment rate (20% versus 8% in the meta-analysis). Retreatment gives moderate pain relief (overall pain response rates of 45–58%) regardless of prior response to palliative radiotherapy [4].

Palliative radiation therapy for bone metastases is usually performed using conventional or at most 3D-conformal radiotherapy (3D-CRT), rather than more advanced techniques such as intensity-modulated radiotherapy (IMRT). As a result, these palliative patients can sometimes suffer from rather pronounced acute toxicities, often during the last months of their lives. Stereotactic body radiotherapy (SBRT) is a recent state-of-the-art form of radiotherapy, typically delivering very high doses (> 6.0 Gy per fraction) in a limited number of fractions (1–5) in a highly conformal manner. This technique is safe due to corresponding improvements in image-guided radiotherapy (IGRT), allowing to continuously monitor the treatment as it is being delivered [5]. SBRT is able to deliver significantly higher biologically equivalent doses (BED) as compared to conventional radiation with improved sparing of surrounding normal tissues. It has consistently demonstrated local control rates of around 90%, even in “radioresistant” tumors [6]. Most studies on SBRT for bone metastases to date focused on so-called “oligometastatic” patients, with only a limited number of (usually asymptomatic) metastases, assuming that ablation of these lesions could result in improved disease-free and perhaps even overall survival [7]. While this is certainly a worth-wile approach, it seems reasonable to also use this technique for palliative patients suffering from painful bone metastases.

The highly conformal delivery of SBRT will hopefully result in an improved acute toxicity profile, based on the experiences captured in patient-reported rather than physician-reported measures. The concept of quality of life (QoL) is subjective; however, in many cancer cohorts, specific tools or patient-reported outcome measures (PROMs) have been developed and validated [8]. These questionnaires assess common issues that affect patients after diagnosis and treatment, and generate scores that reflect the impact on perceptions of health-related quality of life (HRQoL). A secondary aim of this study is to compare hand-written “paper” PROMs to electronic, “paper-less” PROMs.

Moreover, it is now possible to deliver escalated doses to the metastases. We propose a single fraction (avoiding the complexity of multiple fractions) of 20.0 Gy, which has a vastly superior BED compared to the previous (multiple fraction) dose-escalation attempts. It is to be assumed that with these truly “ablative” doses, not only a higher response rate can be achieved but also longer duration of pain control and less re-irradiation need. Perhaps this increased efficacy will compensate the higher treatment cost of SBRT, through less re-treatment and less symptomatic skeletal events (SSEs, consisting of symptomatic pathologic fractures, radiation or surgery to bone, and spinal cord compression).

## Methods/design

### Study design

**Fig. 1:**
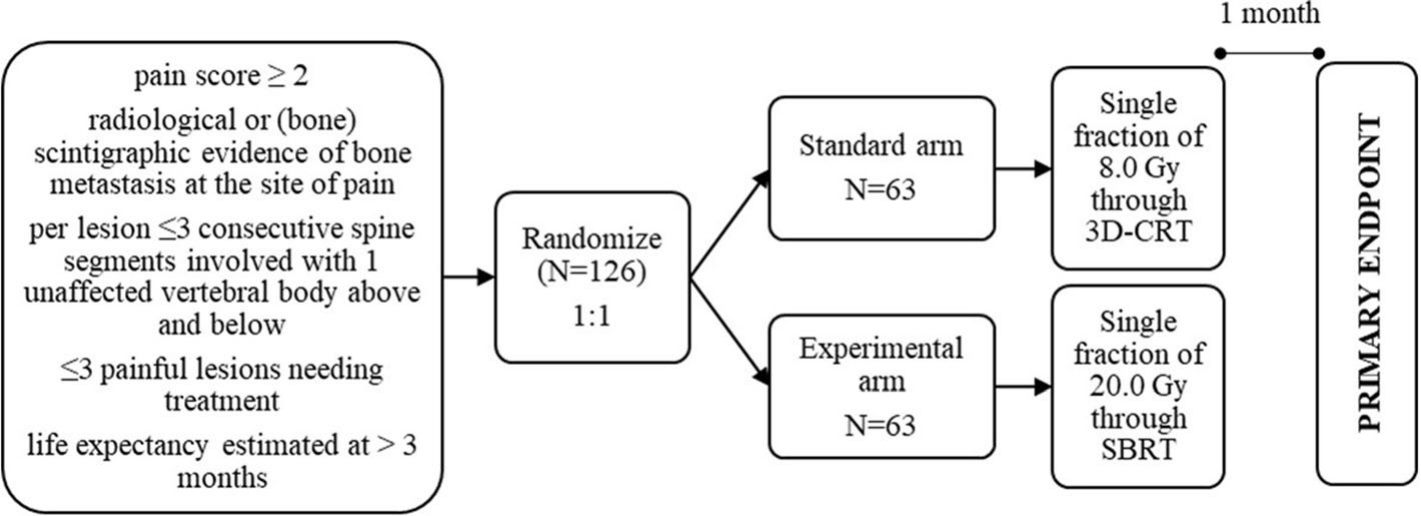
Study schema. Subjects who meet eligibility criteria and qualify for enrolment will be randomized as demonstrated

This is a phase III, randomized-controlled, single-blind, multicenter study comparing the standard schedule for antalgic radiotherapy of a single fraction of 8.0 Gy delivered through 3D-CRT to a single fraction of 20.0 Gy delivered through SBRT (Fig. 1). The primary aim of this trial is to double the complete response rate. Secondary aims are to compare pain flare, duration of pain response, acute and late toxicity, HRQoL through PROMs, re-irradiation need and subsequent SSE.

All subjects will be randomly assigned in a 1:1 ratio to receive either a single fraction of 8.0 Gy to the painful bone metastasis through 3D-CRT (control arm) or a single fraction of 20.0 Gy to the painful bone metastasis through SBRT (experimental arm). A block randomization with a block size of four will be performed by using an electronic randomization tool (Dyco Capture, DigiDyco).

This study has been approved by the Ethics Committee of GZA Hospitals and all collaborating institutions. All patients must provide written informed consent before enrolment. Monitoring will be carried out in this trial.

### Study objectives

#### Primary endpoint

Primary endpoint of this study is pain response at the treated index site at 1 month after RT, as defined according to the International Bone Metastases Consensus Endpoints for Clinical Trials (Table 1) [9].

**Table 1:** Response rate to radiotherapy according to the international consensus [9]

Complete response	Pain score of 0 at the treated site and stable or reduced analgesics in daily oral morphine equivalent (OMED).
Partial response	Pain reduction of 2 or more at the treated site on a scale of 0 to 10 scale without analgesic increase, or analgesic reduction of 25% or more from baseline without an increase in pain.
Pain progression	Increase in pain score of 2 or more above baseline at the treated site with stable OMED, or an increase of 25% or more in OMED compared with baseline with the pain score stable or 1 point above baseline
Indeterminate response	Any response that is not captured by the complete response, partial response, or pain progression definitions

#### Secondary endpoints

Secondary endpoints include pain flare at 24–48-72 h after radiotherapy, the duration of pain response, re-irradiation need, toxicity, HRQoL and subsequent SSE. Pain flare at 24–48-72 h after radiotherapy is defined as pain progression according to the consensus statement [9]. Duration of pain response starts at response until pain progression. Toxicity will be measured with the Common Terminology Criteria for Adverse Events (CTCAE) version 5.0 at 1 month after RT and three-monthly during the first year after treatment. HRQoL is measured by the EORTC QLQ-C30 general questionnaire and the bone metastasis-specific module, the EORTC QLQ-BM22 [10]. Patients fill out these questionnaires before the start of RT (baseline), 1 month after RT (primary endpoint) and three-monthly during the first year after treatment (follow-up). Subsequent SSE is defined as symptomatic pathologic fractures, radiation or surgery to bone, and spinal cord compression.

### Eligibility criteria

Eligible patients are patients with a pain score ≥ 2 on a scale from 0 to 10 (measured as the worst pain for the previous 3 days at the index site), with radiological or (bone) scintigraphic evidence of bone metastasis at the site of pain and no more than 3 painful lesions needing treatment. If analgic dosing adjustment is done less than 1 week before initiation of irradiation, a run-in period is recommended to minimize the risk that the analgesic effects will confound the measurement of the RT effects. Patients should have a life expectancy estimated at > 3 months. Per lesion, no more than 3 consecutive spine segments should be involved, with one unaffected vertebral body above and below. Bone metastasis in previously irradiated sites, or originating from myeloma, or complicated bone metastasis, i.e. impending and/or existing pathological fracture, spinal cord compression or cauda equina compression [11], should be excluded.

### Trial treatments

For patients in the standard arm, the current standard treatment will be prescribed, i.e. a single fraction dose of 8.0 Gy to the metastasis with a planning target volume (PTV) margin for set-up and positioning uncertainties of 1 cm. This can be performed at any linear accelerator.

In the experimental arm, treatment will be delivered within the framework of SBRT. A single fraction dose of 20.0 Gy will be delivered to the metastasis using a PTV margin of 3–5 mm based on high-precision IGRT. Therefore, only linear accelerators with the European Organization for Radiotherapy & Oncology advisory committee on radiation oncology practice (ESTRO-ACROP) specifications for SBRT can be accepted [12]. A risk-adapted approach will be applied, aiming for the highest possible dose no less than 16 Gy, while respecting the tolerances of critical organs at risk (e.g. spinal cord, cauda equina, brainstem etc.).

### Radiotherapy details

#### Simulation and immobilization

Patient immobilization and CT simulation will be done similarly as described in a previous published study protocol from our research group on SBRT for bone metastases [13].

#### Target contouring

The gross tumor volume (GTV) will be delineated as visualized on CT. No clinical target volume (CTV) will be delineated in the experimental arm. In the standard arm standard CTV margins (e.g. incorporating the entire vertebra) are allowed. A planning target volume (PTV) will be created, allowing for daily set-up variance and organ motion. In the standard arm, PTV margins of 1 cm are common, in the experimental (SBRT) arm, PTV margins are 3 to 5 mm.

#### Organs at risk

The organs at risk (OARs) depend on the localization of the metastases. At least all OARs for which dose constraints are described in the report of the American Association of Physicists in Medicine (AAPM) task group 101 [14], lying within the scanned range on the planning CT scan, should be delineated. For spinal lesions, MRI is recommended for spinal cord delineation. A Planning Organ at Risk Volume (PRV) expansion of 2-5 mm will be added to the spinal cord, oesophagus, mediastinum, liver, heart and kidney for setup uncertainty or organ motion. If no MRI is used for delineating the spinal cord, the whole spinal canal should be delineated as PRV. All dose constraints apply to this PRV and should not be exceeded. In case of an overlap of the target with an OAR or PRV, target coverage can be lowered in order to meet the constraint.

#### Treatment planning

In the standard arm, 3D-conformal radiotherapy with basic image-guidance will be used. In the experimental arm, static or rotational treatment planning will be applied depending the localization of the metastasis. Three-dimensional or intensity-modulated coplanar or non-coplanar beam arrangements will be custom designed for each case to deliver highly conformal dose distributions. For high dose-hypofractionated radiotherapy, typically, ≥ 10 beams of radiation are used with roughly equal weighting. When static beams are used, a minimum of 7 beams should be used and non-opposing, non-coplanar beams are preferable. For arc rotation techniques, a minimum of 340 degrees (cumulative for all beams) is warranted.

#### Dose prescription and constraints

In the standard setting, 95% of the PTV should receive 95% of the prescribed dose while near maximum dose (Dnear-max) in the PTV should not exceed 107%. In the experimental arm, treatment will be prescribed to the periphery of the target, i.e. 80% of the dose should cover 95% of the PTV. In the experimental arm, coverage of PTV with the prescribed dose (20Gy) should be optimized to reach 90% or more. Coverage of the PTV with 80% of the prescribed dose (16Gy) should at least reach a minimum of 80% of the PTV with no violations of treatment planning objectives for OAR. Coverage of < 90% of the PTV with 16Gy is a Variation Acceptable, and any coverage of < 80% of the PTV with 16Gy is Deviation Unacceptable. The OAR dose constraints will be in accordance with the recommendations from the report of the AAPM task group 101 [14]. Maximum PTV dose up to 140% is allowed but all dose > 105% should be contained within the GTV. A dose fall-off outside the PTV extending into normal tissue structures should aim at 50% of the prescribed dose within 3 cm.

#### Delivery and verification

In the standard arm, image-guidance will consist of portal images showing the relevant bony anatomy. For the experimental arm, treatment will be delivered with 6–18 MV photons of a linear accelerator with ESTRO-ACROP specifications for SBRT [12]. Image-guidance will consist of cone-beam CT in combination with 6 degrees of freedom corrections using robotic couch. No other RT than photon therapy is permitted. The same position and immobilization/support device(s) as used in the planning CT scan should be utilized. For the investigational arm, a pre-treatment patient-specific and treatment verification quality assurance (QA) program based on transmission dose measurements and cone-beam CT data will be performed.

#### Treatment compliance

Radiotherapy dosing and delivery will be assessed by coverage and dose on target volumes GTV, CTV (if used) and PTV and must be captured in the source documents and the eCRF.

### Interventions

The screening procedures will determine subject eligibility according to inclusion and exclusion criteria. The following evaluation/assessments will be performed at the screening visit within 20 days of Day 1 (Table 2):

- Obtain informed consent
- Record the numeric pain rating scale
- Record the daily oral morphine equivalent (OMED) and other analgesics
- Record education level and computer experience
- Collect details of disease and concurrent systemic anticancer treatment
- ECOG performance status
- HRQoL questionnaires (and reason for non-compliance, if applicable)
- Toxicity (using the most recent version of CTCAE)

**Table 2:** Trial flowchart

	Screening	Treatment					Follow-up
		Simulation	During study				Year 1
			RT	24–48-72 h	weekly	1 m	Every 3 m
Informed consent	x						
Registration of education level, computer experience^a^	x						
Randomization: 1 × 8.0 Gy 1 × 20.0 Gy		x					
Numeric Pain Rating Scale	x		x	x	x	x	x
ECOG performance status	x		x			x	
OMED + other analgesics	x		x	x	x	x	x
Registration of toxicity						x	x
Registration of QoL^b^	x					x	x
Registration of treatment data^c^			x				

^a^Education level:highest level of education (none, primary school, secundary school or higher education); computer experience: at least once a week access to computer/e-mail (yes or no)

^b^QoL according to the EORTC QLQ-C30 & BM22 questionnaires; if form is not completed, reason for non-compliance will be documented in compliance form

^c^See Section “Treatment compliance”

At Day 1, randomization and computed tomography (CT) simulation will be performed.

The following assessments must occur on the day of radiotherapy treatment (Table 2):

- Record the numeric pain rating scale
- Record the daily oral morphine equivalent (OMED) and other analgesics
- Collect RT dosing and delivery details
- ECOG performance status
- HRQoL questionnaires (and reason for non-compliance, if applicable)
- Toxicity (using the most recent version of CTCAE)

At 24 h, 48 h, 72 h, 1 week (+/− 3 days), 2 weeks (+/− 3 days) and 3 weeks (+/− 3 days) after RT subjects are asked to rate their pain flare and record concurrent medications in a pain diary.

The following procedures are to be conducted at primary endpoint visit (1 month after RT) and at each follow-up visit every 3 months up to 12 months (Table 2):

- Record the numeric pain rating scale
- Record the daily oral morphine equivalent (OMED) and other analgesics
- Record the need for re-irradiation
- Record the presence of a symptomatic skeletal event
- Determine the pain response
- ECOG performance status
- HRQoL questionnaires (and reason for non-compliance, if applicable)
- Toxicity (using the most recent version of CTCAE)

### Statistical analysis

#### Sample size calculation

Currently, a complete pain response rate of maximum 25% after a single fraction of 8.0 Gy can be assumed [1–3]. With 116 patients, we can show a statistically significant increase to 50% in complete pain response (with Type I error of 0.05 and power of 0.8). Assuming a drop-out rate of 10%, we need to include 126 patients.

#### Data analysis

All data will be prospectively collected. Electronic case report forms will be used. Statistics will be carried out using the latest version of R.

### Safety

Suspected unexpected serious adverse reactions (SUSARs) that result in death or are life threatening will be reported to the minister and the competent ethics committee within 7 days. All other SUSARs will be reported within 15 days following notification. Once a year throughout the experiment, an annual safety report shall be provided to the ethics committee, listing all suspected serious adverse reactions which have occurred over this period, as well as a report on the safety of the participants. Regarding those adverse events and serious adverse reactions the Principal Investigator will take all reasonable measures to protect subjects at risk following the occurrence of such events. Compensation for any damages incurred by a study patient and linked directly or indirectly to the participation to the study is provided through insurance.

### Supplementary analysis

It is well established that patient and clinician symptom reports are discrepant, with clinicians generally underreporting the incidence and magnitude of symptoms compared with patients [15]. Patient-reported outcome questionnaires assess topics a patient can report about his or her own health, including symptoms, physical functioning, and mental health. Patients report this information via questionnaires that have been rigorously developed. Patient-reported outcome measures (PROM’s) assessed in cancer randomised controlled trials provide valuable information on the impact of treatment from the patient’s perspective. There is even evidence that using PROM’s in palliative oncological patients improves overall survival [16].

Yet, shortcomings in PROM’s trial design, methodology and reporting may limit the interpretation of these data. When patient responses are utilized as measures of primary and secondary endpoints, completion of required assessments is necessary to draw proper conclusions [17]. Efforts should be made to ensure patient-compliance, in order to provide complete datasets. Non-compliance with planned questionnaires and missing data can threaten both internal validity and generalizability. Administrative failure is one of the most important factors leading to non-compliance, against others like patients age [18].

Using electronic PROM’s has some important advantages over using paper-and-pencil questionnaires, e.g. reducing missing data within one assessment, implementing complex skip patterns, eliminating ambiguous data, reducing effort and error in entering data, registering response time, and real-time monitoring of PRO, to name a few. A study evaluating the impact of collecting PROM’s electronically showed greater benefits for computer-inexperienced patients, who were overall older, frailer, and more symptomatic than computer-experienced patients [16]. Participants lacking computer experience may have less-developed health communication skills and thereby benefit more from a structured program for eliciting symptoms. A negative effect of collecting PROM’s electronically is that this reduces physical communication and interaction between the patient and the medical staff.

As a supplementary analysis, we will compare patient-compliance for a paper-and-pencil with an electronic mode. Multiple studies support the between-mode equivalence of paper-and-pencil and electronic PROM’s [19, 20]. For the first 63 patients, a booklet with questionnaires (pain diary, QLQ-C30, BM22) will be presented at the visits as defined per protocol. For the last 63 patients, a smartphone app will be installed on the patient’s own smart device, in order to complete the same questionnaires electronically. During each visit, the reason for non-compliance will be documented when a patient does not complete any part of a questionnaire as required per protocol.

Logistic regression techniques will be employed to determine if any patient characteristic (e.g. socio-economic status, educational level, migration background) or clinical events influenced patient compliance.

## Discussion

In this report, we present the rationale and design of the ROBOMET trial, a randomized study in radiotherapy for painful bone metastases investigating whether SBRT can increase the pain response while at the same time limit the side-effects. It is clear that, although palliative antalgic radiotherapy is an established treatment for painful bone metastases, there is important room for improvement, both regarding efficacy as well as toxicity. Many costly bone-targeted therapies such as osteoclast inhibitors as well as radiopharmaceutical agents have been developed, but palliative radiotherapy remains the mainstay for local treatment and symptom control. It is therefore to be expected that this patient-directed trial can improve the quality of life of a great number of cancer patients worldwide on short term.

One important measure to improve pain response to radiotherapy would be to escalate the dose delivered to the tumor. Evidence for this emerges from multiple prospective studies. Researchers from Ghent University Hospital randomized (1,1:1) 45 patients with uncomplicated painful bone metastases to receive either 8.0 Gy in a single fraction with conventional radiotherapy (arm A) or 8.0 Gy in a single fraction with dose-painting by numbers IMRT up to 10.0 Gy (arm B) or 16.0 Gy in a single fraction with dose-painting by numbers IMRT up to 18.0 Gy (arm C). The primary endpoint was overall pain response at 1 month. Eight (53%), 12 (80%) and 9 patients (60%) had an overall response to treatment in arm A, B and C, respectively [21]. In an American single-institute series, 49 patients with 61 separate spinal metastases were treated to a single fraction of 10.0 to 16.0 Gy [22]. Encouragingly, complete pain relief was achieved in 46%, partial relief in 18.9%, and stable symptoms in 16.2% of patients. These data suggest that a single dose-escalated fraction could result in complete pain response rates of around 50%.

A major advantage of SBRT over 3DCRT is an expected reduction in dose to the normal tissue, which presumably will lead to less acute toxicity. Already numerous case series and multiple prospective trials have proven that both multi- and single fraction SBRT schedules for bone metastasis can be delivered with minimal toxicity [23–27]. Especially in a palliative setting, even transient side effects like nausea and diarrhoea are cumbersome. In this regard, optimisation of palliative radiation treatment through the use of SBRT is one of the measures that should be investigated, because quality of life is for most of these patients of ultimate priority.

On the other side, some reports indicate that high dose single fraction SBRT is associated with a greater incidence of pain flare. In prospective studies using 3DCRT for painful bone metastases, the incidence of pain flare is approximately 40% [28, 29] whereas this incidence ranges between 10 and 68% after SBRT [30–34]. One explanation for this large range is the difference in fractionation schedules that are used, with a single fraction SBRT potentially leading to more pain flare. Besides that, inconsistency in the definitions of pain flare, the use of retrospective data, administering corticosteroids in the prevention of pain flare, or physicians rather than patients reporting pain scores, are other factors making it difficult to compare between these results.

A potential disadvantage of SBRT could be the higher rate of vertebral compression fractures (VCF) associated with this technique. At least in the spine, the use of high dose single fraction SBRT might be associated with a higher risk of vertebral compression fractures. In a large multi-institutional investigation of spine SBRT related VCF, a dose-complication relationship was observed based on the dose-per-fraction. A 39% risk of VCF was observed with high dose single fraction SBRT (≥24 Gy), 23% with a dose per fraction of 20 to 23 Gy, and 11% when below 20 Gy [35]. In order to identify patients who are stable, potentially unstable or frankly mechanically unstable, the Spinal Instability Neoplastic Score (SINS) was developed [36], which incorporates several of the significant predictive factors on either uni- or multivariate analysis of trials evaluating VCF after SBRT [37]. Most VCF are observed shortly after SBRT, with a median time to VCF of 2.6 months according to the systematic review of Faruqi e.a [37]. However, in the calculation of this median time to VCF, a study with a median of 25 months was treated as outlier and excluded [38]. In order to evaluate the incidence of VCF in our patient cohort, serious SSE will be evaluated until 1 year after completion of treatment, which we believe will capture most of the treatment-related VCF’s.

To our knowledge, there are no published randomized trials comparing conventional to stereotactic radiotherapy in polymetastatic cancer patients with bone metastases, but multiple other trials have been initiated to look at the efficacy and safety of SBRT for painful (spinal) bone metastases. The American RTOG-0631 trial aimed to randomize (1:2) 240 patients with localized spinal metastases between a single conventional RT fraction of 8.0 Gy vs. a single SBRT fraction of 16.0 or 18.0 Gy (with dose as preferred by the treating physician). Primary endpoint is complete or partial pain relief at the treated index site at 3 months. Accrual has recently finished and results are awaited [39]. This trial is clearly very similar but focussed exclusively on spinal metastases, where the dose is limited due to the proximity of the spinal cord. Another trial is the Dutch VERTICAL trial, aiming to randomize (1:1) 110 patients with painful bone metastases to either between a single conventional RT fraction of 8.0 Gy vs. a single MRI-based SBRT fraction of 18.0 Gy to the visible metastasis and 8.0 Gy to the bony compartment containing the metastasis. Primary endpoint is complete or partial pain response at 3 months after radiotherapy. This trial is currently still recruiting [40]. Recently, the results of a randomized trial of the Heidelberg University were published. In this trial, 55 patients with painful spinal metastases were treated with either single fraction SBRT (24Gy) or 3DCRT (30Gy in 10 fractions). The trial demonstrated that single-fraction SBRT reduced pain levels faster during the 3 months following RT and led to improved pain scores compared to 3DCRT.

Currently, the innovations (IMRT, IGRT) that have fuelled many of the significant advances in curative radiotherapy are not sufficiently being applied for palliative indications. This is partly due to limited resources, since these new techniques take up more time on the treatment machine and also more extensively occupy health care providers, both physician, physicist and radiation therapist, compared to conventional radiotherapy. However, by minimizing subsequent re-irradiation, reducing pain medication and preventing costly SSEs, this new technique can have a favourable socio-economic impact. Therefore, randomized evidence supporting the utility of advanced technologies in the palliative setting will be needed to convince both the radiation oncology community as well as the relevant governments and reimbursement agencies of the need to apply these innovative techniques to palliative patients.

## Data Availability

Data sharing is not applicable to this article as this trial is ongoing.

## Abbreviations

3D-CRT: Three-dimensional, conformal radiotherapy
AAPM: American Association of Physicists in Medicine
ACROP: Advisory committee on radiation oncology practice
AE: Adverse event
AR: Adverse reaction
BED: Biologically equivalent dose
CT: Computed tomography
CTCAE: Common terminology criteria for adverse events
CTV: Clinical target volume
EBRT: External-beam radiotherapy
EC: Ethics committee
eCRF: Electronic case report form
EORTC: European Organisation for Research and Treatment of Cancer
ESTRO: European Organization for Radiotherapy & Oncology
GTV: Gross tumor volume
HRQoL: Health-related quality of life
IC: Informed consent
IGRT: Image-guided radiotherapy
IMRT: Intensity-modulated radiotherapy
MRI: Magnetic resonance imaging
NPRS: Numeric pain rating scale
OAR: Organs at risk
OMED: Daily oral morphine equivalent
PROM: Patient-reported outcome measure
PRV: Planning organ at risk volume
PTV: Planning target volume
QA: Quality assurance
QLQ: Quality of life questionnaire
QoL: Quality of life
RTOG: Radiation Therapy Oncology Group
SAE: Serious adverse event
SAR: Serious adverse reaction
SBRT: Stereotactic body radiotherapy
SINS: Spinal instability neoplastic score
SSE: Symptomatic skeletal events
SUSAR: Suspected unexpected serious adverse reaction
VCF: Vertebral compression fracture
